# Performance of the Pandemic Medical Early Warning Score (PMEWS), Simple Triage Scoring System (STSS) and Confusion, Uremia, Respiratory rate, Blood pressure and age ≥ 65 (CURB-65) score among patients with COVID-19 pneumonia in an emergency department triage setting: a retrospective study

**DOI:** 10.1590/1516-3180.2020.0649.R1.10122020

**Published:** 2021-03-03

**Authors:** Mehmet Cihat Demir, Buğra Ilhan

**Affiliations:** I MD. Assistant Professor, Department of Emergency Medicine, Düzce University School of Medicine, Düzce, Turkey.; II MD. Attending Emergency Physician, Department of Emergency Medicine, University of Health Sciences, Bakırköy Dr. Sadi Konuk Training and Research Hospital, Istanbul, Turkey.

**Keywords:** COVID-19 [supplementary concept], Emergency service, hospital, Pandemics, Pneumonia, Triage, CURB-65, PMEWS, STSS, Triage scores.

## Abstract

**BACKGROUND:**

Healthcare institutions are confronted with large numbers of patient admissions during large-scale or long-term public health emergencies like pandemics. Appropriate and effective triage is needed for effective resource use.

**OBJECTIVES:**

To evaluate the effectiveness of the Pandemic Medical Early Warning Score (PMEWS), Simple Triage Scoring System (STSS) and Confusion, Uremia, Respiratory rate, Blood pressure and age ≥ 65 years (CURB-65) score in an emergency department (ED) triage setting.

**DESIGN AND SETTING:**

Retrospective study in the ED of a tertiary-care university hospital in Düzce, Turkey.

**METHODS:**

PMEWS, STSS and CURB-65 scores of patients diagnosed with COVID-19 pneumonia were calculated. Thirty-day mortality, intensive care unit (ICU) admission, mechanical ventilation (MV) need and outcomes were recorded. The predictive accuracy of the scores was assessed using receiver operating characteristic curve analysis.

**RESULTS:**

One hundred patients with COVID-19 pneumonia were included. The 30-day mortality was 6%. PMEWS, STSS and CURB-65 showed high performance for predicting 30-day mortality (area under the curve: 0.968, 0.962 and 0.942, respectively). Age > 65 years, respiratory rate > 20/minute, oxygen saturation (SpO_2_) < 90% and ED length of stay > 4 hours showed associations with 30-day mortality (P < 0.05).

**CONCLUSIONS:**

CURB-65, STSS and PMEWS scores are useful for predicting mortality, ICU admission and MV need among patients diagnosed with COVID-19 pneumonia. Advanced age, increased respiratory rate, low SpO_2_ and prolonged ED length of stay may increase mortality. Further studies are needed for developing the triage scoring systems, to ensure effective long-term use of healthcare service capacity during pandemics.

## INTRODUCTION

Coronavirus disease 2019 (COVID-19), which is caused by severe acute respiratory syndrome coronavirus-2 (SARS-CoV-2), first appeared in several pneumonia cases of unknown etiology in Wuhan, China, in December 2019.^1^^,^^2^ It was declared to be a pandemic by the World Health Organization (WHO) because it affected all countries within a short time. COVID-19 infection has been reported over a broad clinical spectrum, ranging from mild symptoms to severe acute respiratory distress syndrome, multiple organ dysfunction syndrome or even death.^3^ Traditional public health measures have been found to be inadequate for preventing it^4^ and no disease-specific treatment or vaccine has yet become available. According to the World Health Organization, more than 18 million cases and approximately 700,000 deaths have been reported.^5^

The increasing numbers of COVID-19 cases worldwide places a heavy burden on healthcare systems in many countries.^6^ Healthcare institutions are suddenly confronted with large numbers of patient applications during large-scale or long-term public health emergencies such as pandemics. Moreover, such situations may cause difficulties with regard to medical resources and workforces.^7^

For this reason, it is essential to make the best use of opportunities in pandemics. During a pandemic, the most crucial issue consists of efficient use of capacity with good coordination in response to increasing demand.^8^ The best way to use resources effectively during a pandemic is to conduct appropriate triage.^9^ Since it is impossible to know when the next pandemic will occur, the problems experienced during current pandemic periods need to be defined. In addition, the most appropriate triage method should be determined. There is a need for objective and reliable scoring systems that can predict disease progression at the time when patients arrive and guide physicians to decide whether to admit or discharge them.

The Confusion, Uremia, Respiratory rate, Blood pressure and age ≥ 65 years (CURB-65) score has been used as a safe predictor of 30-day mortality among patients with pneumonia for many years.^10^ It also helps clinicians in making the decision to admit or discharge such patients.

The Pandemic Medical Early Warning Score (PMEWS), developed by Challen et al. for use in cases of influenza pneumonia, evaluates the patients’ social and physiological parameters.^11^ This pandemic score was developed by adding age, social isolation, chronic disease and performance status to the patient’s vital parameters. In a comparison between the PMEWS and the CURB-65 score, the PMEWS performed better regarding assessment of the need for intensive care unit (ICU) admission and hospitalization, but lagged behind the CURB-65 score for predicting mortality.^11^

Talmor et al. developed the Simple Triage Scoring System (STSS) with the aims of enabling efficient resource use and identifying critically ill patients during a pandemic, but stated that it still needed modification and validation.^12^ Adeniji et al. reported that the STSS could predict mortality and the use of critical care resources in situations of pandemic influenza.^13^

Like in the CURB-65 score, age, altered mental status and vital parameters are used in the STSS. Unlike in the CURB-65 score, no laboratory results are used either in the PMEWS or in the STSS. The different characteristics of these scoring systems enable evaluation of patients and fast decision-making in the triage setting, regarding admission.

## OBJECTIVE

To our knowledge, there are not enough studies on triage scores relating to COVID-19. The aim of this study was to evaluate the performance of the Pandemic Medical Early Warning Score, Simple Triage Scoring System and Confusion, Uremia, Respiratory rate, Blood pressure and age ≥ 65 score for predicting intensive care unit admission, mechanical ventilation (MV) need and 30-day mortality among patients with COVID-19 pneumonia, and whether these are useful scoring systems.

## METHODS

### Study design

This was a retrospective and observational study. After obtaining approval from the local ethics committee (approval ID: 2020/85; June 1, 2020), patients diagnosed with COVID-19 pneumonia in our hospital’s emergency department (ED) between March 11, 2020, and June 11, 2020, were included. Patient data were obtained from the hospital’s electronic database and from the records of the hospital’s ED.

This study was conducted in the ED of a tertiary-level university hospital, which receives approximately 75,000 patient admissions annually. The patients were followed up in six-bed pandemic tents or in two-bed isolation rooms within the 15-bed emergency department, and sampling was carried out there. On admission, swab samples were taken from the nasopharynx and oropharynx using the same stick.

Demographic information (age, gender and comorbidities), smoking status, complaints at the time of admission, vital values on admission (fever, pulse, respiratory rate, blood pressure, oxygen saturation and shock index), computed tomography results, emergency department and hospital length of stay (LOS), 30-day mortality, mechanical ventilation need and ICU admission were recorded on the study forms. The CURB-65, PMEWS and STSS scores were calculated.

### Participants and measurements

Adult patients over 18 years old with real-time polymerase chain reaction (RT-PCR) positivity in samples taken to detect COVID-19 infection were included (n = 111). Patients whose data could not be accessed (n = 3), those referred by another center (n = 5) and those diagnosed with COVID-19 in another clinic (n = 3) were excluded. For patients with more than one RT-PCR test, the results from the tests done on admission were evaluated. Swab samples taken using plastic-coated sticks were sent to the laboratory in a viral transport medium. PCR testing was performed using a SARS-CoV-2 quantitative RT-PCR detection kit (Bioeksen R&D Technologies, Istanbul, Turkey) and Montania RT-PCR instruments (Anatolia Geneworks, Istanbul, Turkey).

Other studies in the literature^12^^,^^13^ were used to determine the minimum sample size to be included in the present study. Accordingly, the minimum number of individuals, sampled at 80% statistical power with a 95% confidence interval and 5% type 1 error, was determined as 100.

In determining the CURB-65 score, each of the following parameters was calculated as one point: presence of confusion, blood urea nitrogen (BUN) > 19 mg/dl, respiratory rate ≥ 30 breaths/minute, systolic blood pressure < 90 mmHg or diastolic blood pressure ≤ 60 mmHg and age ≥ 65 years.^10^ The CURB-65 score can range from 0 to 5 and was interpreted thus: patients with scores of 0 or 1 point can be discharged; 2 points should be admitted or kept under observation for a while; and ≥ 3 points must be admitted and the need for ICU admission must be considered.

For PMEWS, respiration rate, oxygen saturation, heart rate, systolic blood pressure, fever, consciousness level, age, social isolation, history of chronic disease and daily activity performance status were examined.^11^ The patients’ physiological values are scored between 0 and 3. However, for patients older than 65 years, one additional point is given, and there is one further point for social isolation status or chronic disease, or for performance status > 2.

The STSS score was calculated from the patients’ vital parameters, shock index and age. The presence of respiratory rate > 30 breaths/minute, shock index (heart rate/systolic blood pressure) > 1, low oxygen saturation (SpO_2_ < 90%), altered mental status (Glasgow Coma Scale, GCS < 15) and age > 65 years were each scored as one point.^12^ The scores were interpreted thus: 0 or 1 point represented a mild risk of mortality; 2 points, medium risk; and ≥ 3 points, high risk.

### Outcome

The primary outcomes of this study were the patients’ performances relating to all three scores, for predicting all-cause 30-day mortality, ICU admission and mechanical ventilation need. The secondary outcomes were the effects from the patients’ vitality on admission, demographic characteristics and hospital emergency department length of stay, in relation to mortality.

### Data analysis

Descriptive statistics were presented as numbers and percentages. Numerical variables were summarized as the mean ± standard deviation or median (with interquartile range). The independent t test and Mann-Whitney U test were used to compare the groups according to distributions. The receiver operating characteristic (ROC) curve and the area under the ROC curve (AUC) with 95% confidence intervals were used to assess the accuracy of each score. Pearson’s chi-square test and Fisher’s exact test (when the expected number was less than five) were used for categorical variables. The statistical analyses were performed using the SPSS software for Windows, version 22 (IBM, Chicago, IL, United States). P < 0.05 was considered significant.

## RESULTS

In total, 100 patients were included in the study. The patients’ median age was 50.78 ± 16.75, and 54% of the patients were women. The RT-PCR results were positive for all the patients included in this study. The mortality rate among the patients included in this study was 6%, and all of the patients who died had been admitted to the intensive care unit. The mortality rates among patients with comorbid diseases and mechanical ventilator need were significantly higher (P < 0.05). Thoracic computed tomography imaging was performed on 90% of the patients. Mortality was not observed among the patients who did not undergo computed tomography imaging because their complaints were mild (n = 10), or among the patients whose imaging examinations did not show any pathological findings (n = 13). The distribution of the patients’ clinical characteristics, symptoms, pathological computed tomography (CT) findings, mechanical ventilation need and hospitalization/discharge outcomes, in relation to 30-day mortality, is shown in Table 1.

**Table 1.** Distribution of patients’ clinical characteristics, symptoms, pathological computed tomography findings, mechanical ventilation need and hospitalization/discharge outcomes, in relation to 30-day mortality

	n = 100	30-day mortality		P
		Yes	No	
**Female**	54	4	50	0.684
**Comorbid disease**	43	6	37	**0.005**
**Smoking habit**	19	1	18	1.0
**Symptoms**				
Fever	29	4	25	0.057
Cough	52	4	48	0.679
Dyspnea	15	2	13	0.220
Myalgia	15	1	14	1.0
Sore throat	7	0	7	1.0
Diarrhea	2	1	1	0.117
Loss of taste	3	0	3	1.0
Loss of smell	1	0	1	1.0
Headache	2	0	2	1.0
Asymptomatic	24	0	24	0.331
**Pathological CT findings**	77	6	71	0.385
**Need for MV**	15	6	9	**< 0.001**
**Hospitalization/discharge outcomes**				
Discharge	40	0	40	**< 0.001**
Ward admission	50	0	50	
ICU admission	10	6	4	

CT = computed tomography; MV = mechanical ventilation; ICU = intensive care unit.

P < 0.05 was considered statistically significant.

In evaluating vital factors on admission, significant relationships with mortality were only found in relation to respiratory rate and oxygen saturation (P < 0.05). No significant relationship was found between mortality and other vital factors. There were significant relationships between advanced age and mortality and between prolonged emergency department length of stay and mortality (p < 0.05). Advanced age (> 65 years), increased respiratory rate (> 20/minutes), low SpO_2_ (< 90%) and prolonged ED length of stay (> 4 hours) significantly increased mortality (P = 0.02, P = 0.02, P < 0.001 and P = 0.02, respectively). The relationships of patients’ age, vital factors on admission, shock index and emergency department and hospital length of stay with mortality are shown in Table 2.

**Table 2.** Factors affecting mortality among COVID-19 pneumonia patients

	30-day mortality		P	All patients
	Yes	No		
Age^a^ (year)	72.67 ± 15.73	49.38 ± 15.89	0.001^*^	50.78 ± 16.75
SBP^b^ (mmHg)	114.5 (89.5-141.5)	125 (117.75-140)	0.225^**^	125 (116.25-139.75)
DBP^a^ (mmHg)	66.5 ± 13.47	75.83 ± 13.56	0.105^*^	75.27 ± 13.67
Heart rate^b^ (beats/min)	90 (78.25-112.5)	88 (78.75-99.5)	0.566^**^	88 (79.0-100.5)
Respiratory rate^b^ (breaths/min)	20 (14-27)	14 (14-16)	0.028^**^	14 (14-16)
SpO_2_^b^ (%)	86.5 (57.5-93.25)	96 (94-98)	0.002^**^	96 (94-98)
Fever^b^ (°C)	36.15 (36.07-38.72)	36.8 (36.27-37.4)	0.541^**^	36.8 (36.2-37.4)
Shock index^b^	0.74 (0.65-1.1)	0.67 (0.59-0.77)	0.117^**^	0.67 (0.60-0.77)
LOS in ED^b^ (hour)	5.5 (2.75-6.25)	3 (2-4)	0.039^**^	3.0 (2-4)
LOS in hospital^b^ (day)	6 (2.75-11)	4.5 (0-8)	0.186^**^	4.5 (0-8)

^a^mean ± standard deviation; ^b^median (interquartile range); ^*^independent t test; ^**^Mann-Whitney U test.

SBP = systolic blood pressure; DBP = diastolic blood pressure; SpO_2_ = oxygen saturation; LOS = length of stay; ED = emergency department; P < 0.05 was considered statistically significant.

Significant relationships were found between all the PMEWS, CURB-65 and STSS scores and mortality, intensive care unit admission and mechanical ventilation need (P < 0.001). The mortality rates were found to be significantly higher when these scores were higher. These relationships are shown in Table 3.

**Table 3.** Comparison of the Pandemic Medical Early Warning Score (PMEWS), Confusion, Uremia, Respiratory rate, Blood pressure and age ≥ 65 (CURB-65) score and Simple Triage Scoring System (STSS) score, in relation to mortality, intensive care unit (ICU) admission and need for mechanical ventilation (MV)

	30-day mortality		P	ICU admission		P	Need for MV		P
	Yes	No		Yes	No		Yes	No	
**PMEWS**									
0-3	0	77	< 0.001^*^	1	76	< 0.001^*^	5	72	< 0.001^*^
4	1	6		1	6		1	6	
5	0	4		0	4		0	4	
6	0	3		1	2		1	2	
7	1	2		2	1		3	0	
8	1	2		2	1		2	1	
12	1	0		1	0		1	0	
13	1	0		1	0		1	0	
14	1	0		1	0		1	0	
**CURB-65**									
0	0	62	< 0.001^*^	1	61	< 0.001^*^	2	60	< 0.001^*^
1	1	23		1	23		3	21	
2	2	7		4	5		6	3	
3	1	2		2	1		2	1	
4	2	0		2	0		2	0	
**STSS**									
0	0	66	< 0.001^*^	1	65	< 0.001^*^	3	63	< 0.001^*^
1	1	22		3	20		5	18	
2	1	5		2	4		2	4	
3	2	1		2	1		3	0	
4	2	0		2	0		2	0	

^*^P < 0.05 was considered statistically significant.

Performance in predicting 30-day mortality was high, through using all three scores. The areas under the curve for PMEWS, CURB-65 and STSS were 0.968, 0.942 and 0.962, respectively. The sensitivity was 100% and the specificity was 81% for PMEWS ≥ 3. The sensitivity was 83% and the specificity was 93% for STSS ≥ 1.

Performance in predicting ICU admissions was high, through using all three scores. The areas under the curve for PMEWS, CURB-65 and STSS were 0.941, 0.898 and 0.878, respectively. The PMEWS had the highest result. The sensitivity was 80% and the specificity was 95% for PMEWS ≥ 5. The STSS score sensitivity was 90% and the specificity was 72% for predicting ICU admission.

Performance in predicting mechanical ventilation needs was high, through using all three scores. The areas under the curve for PMEWS, CURB-65 and STSS were 0.854, 0.867, and 0.820, respectively. The specificities for PMEWS ≥ 5 and CURB-65 ≥ 1 were 96% and 95%, respectively. The sensitivity for STSS ≥ 1 was 80%.

**Figure 1.**
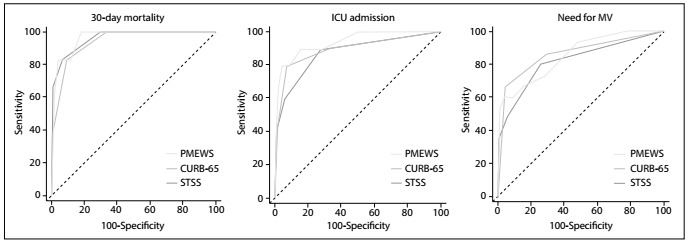
Receiver operating characteristic (ROC) curves for the Pandemic Medical Early Warning Score (PMEWS), Confusion, Uremia, Respiratory rate, Blood pressure and age ≥ 65 (CURB-65) score and Simple Triage Scoring System (STSS) score for 30-day mortality, intensive care unit (ICU) admission and need for mechanical ventilation (MV).

The performance of the scoring systems in predicting mortality, ICU admission and mechanical ventilation needs, in terms of their sensitivity, specificity, positive and negative predictive values and positive and negative likelihood ratios, are shown in Table 4. The receiver operating characteristic curves of the scores are shown in Figure 1.

**Table 4.** Performance, sensitivity, specificity, likelihood ratios and predictive values of PMEWS, CURB-65 and STSS scores

	AUC	95% CI	Sensitivity (%)	Specificity (%)	+LR	−LR	PPV	NPV	P
Mortality									
PMEWS ≥ 3	0.968	0.912-0.993	100	81.91	5.53	0.0	26.08	100.0	< 0.001^*^
CURB-65 ≥ 1	0.942	0.877-0.979	83.33	90.43	8.70	0.18	35.72	98.83	< 0.001^*^
STSS ≥ 1	0.962	0.903-0.990	83.33	93.62	13.06	0.18	45.46	98.87	< 0.001^*^
ICU admission									
PMEWS ≥ 5	0.941	0.875-0.978	80	95.56	18	0.21	66.68	97.72	< 0.001^*^
CURB-65 ≥ 1	0.898	0.821-0.949	80	93.33	12	0.21	57.13	97.67	< 0.001^*^
STSS ≥ 1	0.878	0.798-0.935	90	72.22	3.24	0.14	26.46	98.48	< 0.001^*^
Need for MV									
PMEWS ≥ 5	0.854	0.769-0.917	60	96.47	17	0.41	74.99	93.18	< 0.001^*^
CURB-65 ≥ 1	0.867	0.785-0.927	66.67	95.29	14.17	0.35	71.4	94.18	< 0.001^*^
STSS ≥ 1	0.820	0.731-0.890	80	74.12	3.09	0.27	35.29	95.45	< 0.001^*^

PMEWS = Pandemic Medical Early Warning Score; CURB-65 = Confusion, Uremia, Respiratory rate, Blood pressure and age ≥ 65; STSS = Simple Triage Scoring System; AUC = area under the curve; CI = confidence interval; +LR = positive likelihood ratio; -LR = negative likelihood ratio; PPV = positive predictive value; NPV = negative predictive value; ICU = intensive care unit; MV = mechanical ventilation.

^*^P < 0.05 was considered significant.

## DISCUSSION

It is essential to differentiate severe cases from mild cases at an early stage in situations of major global health problems such as pandemics. Effective triage on admission will prevent unnecessary hospitalizations and resource use.

We concluded that the PMEWS, CURB-65 and STSS scores successfully predicted patients’ 30-day mortality, ICU admissions, and mechanical ventilation needs (P < 0.001). While the sensitivity for mortality shown by scores of PMEWS ≥ 3, STSS ≥ 1 and CURB-65 ≥ 1 were 100%, 83.3% and 83.3%, respectively, their specificities were 81.91%, 93.62% and 90.43% (P < 0.001). Accordingly, scores ≥ 3 from PMEWS were most sensitive for triage, whereas STSS ≥ 1 was most specific in predicting mortality among patients with COVID-19 pneumonia. The areas under the curve for PMEWS ≥ 3, STSS ≥ 1 and CURB-65 ≥ 1 were determined as 0.968, 0.962 and 0.942, respectively. Therefore, we can say that all three scores can be used with high reliability for triage of COVID-19 pneumonia.

Whereas in our study PMEWS ≥ 3 showed high accuracy for predicting mortality, Ebrahimian et al. recommended that patients should be admitted to hospital when PMEWS ≥ 4.^14^ Gray et al. reported that use of the PMEWS was beneficial in the prehospital period.^15^ Although this score has not been evaluated among COVID-19 patients in any other studies, it has been stated that PMEWS could be used successfully among pandemic influenza patients.^11^ Hence, we can say that it can be used in all triage settings.

In a study by Challen et al., it was concluded that the PMEWS was a better predictor than CURB-65 with regard to admission and critical care needs, but that it lagged behind CURB-65 for predicting mortality.^11^ Although CURB-65 had high sensitivity and specificity in our study, it lagged behind PMEWS and STSS for predicting mortality. In evaluations regarding prediction of ICU admissions, the PMEWS had the most successful result, while the STSS had the lowest.

In forecasting the need for mechanical ventilation, the PMEWS and CURB-65 had high specificity but lower sensitivity. Rosenbaum reported that the numbers of mechanical ventilators and intensive care unit beds were insufficient in the COVID-19 pandemic.^16^ Undoubtedly, accurate prediction of which patients may need mechanical ventilation and ICU admission will contribute towards effective use of resources.

However, the need for serum urea measurement to calculate the CURB-65 score prevents rapid assessment. This situation restricts the use of CURB-65 scores in triage settings. Comparison between PMEWS and STSS shows that they have similar power for predicting mortality. Because STSS is calculated with fewer parameters, it is more practical and faster than PMEWS. We found that STSS could be calculated more easily and more quickly than PMEWS and CURB-65 in emergency room triage settings.

Talmor et al. developed the STSS for use in epidemics, and it was found to effectively predict ICU and mechanical ventilator need.^12^ Those authors also noted that modification of this scale might be required, to incorporate data from a real pandemic situation.^12^ In our study, STSS ≥ 1 predicted mortality, in addition to ICU admission and mechanical ventilator need. Morton et al. stated that STSS ≥ 2 was a perfect predictor for critical care among patients with influenza.^17^

A study by Su et al. among COVID-19 patients found that the cutoff of CURB-65 ≥ 2 had 60% sensitivity and 93.4% specificity for predicting ICU need.^18^ In our study, we obtained similar specificity (93.3%) and higher sensitivity (80%) with a lower cutoff (CURB-65 score ≥ 1).

Evaluation of the relationship between age and mortality among patients diagnosed with COVID-19 pneumonia in our study showed that the ages of the patients who died were significantly higher (P = 0.001). The average age of the patients in our study was found to be 51 years. However, serious outcomes such as COVID-19-associated ICU admission or mortality showed highest prevalence at the ages of 70 and above, regardless of the underlying disease.^19^ In another prospective cohort study, the average age at which death due to COVID-19 occurred was 70.2 years.^20^

In the Middle East respiratory syndrome (MERS) epidemic, advanced age was defined as an independent predictor of mortality.^21^ This can be attributed to age-related defects in T-cell and B-cell functions, inability to control viral replication due to excessive production of type-2 cytokines and prolonged pro-inflammatory responses.^22^ In our study, the average age of the patients who died was 72.6 years, and this age was similar to what had been reported in other studies in the literature. Therefore, advanced age should be considered to be an independent variable associated with poor outcomes for COVID-19 pneumonia patients in triage settings. In all three scoring systems that we used in our study, age was included as a criterion.

Dyspnea is a fundamental cause of emergency visits and admissions relating to COVID-19 pneumonia. In our study, 15% of the cases presented with shortness of breath and 52% with coughing. We found that the patients who died had higher respiratory rates and lower oxygen saturation levels on admission (P = 0.028 and P = 0.002, respectively). In a study by Zhou et al., it was observed that mortality was higher among patients with tachypnea.^23^ In a study by Du et al., it was stated that the respiratory rate in the group that died was significantly higher than that of the survivors, and there was no difference in terms of other vital factors such as heart rate.^20^ In a study by Zangrillo et al., similar to our study, mortality was higher among patients with advanced age and low SpO_2_ values on admission.^24^

In line with data in the literature, we did not find any significant relationship between vital parameters (systolic and diastolic blood pressure, pulse rate and fever) and shock index, except for respiratory rate and SpO_2_ (P > 0.05). Alveolar serous exudation, hyaline membrane formation, inflammatory infiltrations, necrosis of pneumocytes, vascular edema, microthrombus and pulmonary interstitial fibrosis have been detected in COVID-19 patients.^25^ COVID-19 is a systemic disease that primarily injures the vascular endothelium, and if dyspnea is not managed, patients may have multiple organ failure even if they are not in an older age group.^26^ Therefore, oxygen therapy is life-saving for patients with COVID-19 pneumonia who have severe respiratory distress or hypoxia.

In our study, the emergency department length of stay of the patients who died was significantly higher (P = 0.039). Patients with advanced age, comorbid disease and worse clinical conditions on admission are further investigated in the emergency department and are referred for consultations at other clinics.

Additional treatments are given to these patients to stabilize their clinical condition. This situation prolongs patients’ emergency department length of stay. In a study by Sabaz et al., prolongation of emergency department length of stay among critically ill patients was associated with worse consequences and increased mortality.^27^ Moreover, emergency department length of stay significantly prolonged the inpatient length of stay in a study by Liew et al.^28^

In the literature, there is no study comparing the relationship between emergency department length of stay among patients with COVID-19 pneumonia and occurrences of mortality. Therefore, our study provides the first data in the literature showing that emergency department length of stay affects mortality. Further studies are needed with regard to emergency department length of stay and the causes and outcomes of delays among patients with COVID-19 pneumonia.

In our study, no significant relationship was found between the length of stay in hospital and mortality (P = 0.186). In a study by Shao et al., in which 136 in-hospital cardiac arrest patients were evaluated, the length of stay in the hospital was reported to be seven days.^29^ In our study, while the length of stay in hospital in the group that died was six days (interquartile range, IQR: 2.7-11 days), it was 4.5 days (IQR: 0-8) in the group of survivors. Liu et al. reported that patients with severe COVID-19 pneumonia and lymphopenia stayed longer in the hospital.^30^ In another study, it was concluded that there was no significant difference in mortality, with regard to length of stay in the ICU.^23^

The Turkish Ministry of Health has recommended that treatments for patients who are hospitalized due to COVID-19 pneumonia should be completed in the same hospital. In this way, the patients’ isolation is provided safely, and their compliance with the treatment is controlled. This recommendation may cause similar lengths of stay in hospital among patients.

The primary limitation of this study is that it was conducted in a single center, with a study group consisting of patients admitted to the emergency department of a tertiary-level university hospital. The low number of cases in the city where the study was conducted caused the number of patients included in this study to be limited. Extensive multicenter studies are needed for the validation of these scoring systems.

The secondary limitation of this study is that it was based on medical records. It was a cross-sectional analysis with a small number of participants. We included all COVID-19 pneumonia patients who were admitted to the emergency department during the study period, and only a few (n = 11) were then excluded. Patients with positive real-time polymerase chain reaction results were included in the study. The patient group may have been affected by the false positivity and negativity of the reference test. Also, there may have been false-negative results, depending on the sampling technique and the region sampled.

## CONCLUSION

The Pandemic Medical Early Warning Score, Simple Triage Scoring System and Confusion, Uremia, Respiratory rate, Blood pressure and age > 65 score can be used safely in triage settings, to determine the prognosis for patients diagnosed with COVID-19 pneumonia. Furthermore, these scores can be used for predicting mortality, ICU admission and mechanical ventilation need. These scores can help in managing resources effectively during a pandemic period. Advanced age, high respiratory rate and low SpO_2_ values significantly increased the mortality among COVID-19 pneumonia patients. Prolonged emergency department length of stay increases mortality. Especially in pandemics, there is a need to apply objective and reliable triage scoring systems that have been verified through comprehensive studies.
